# A Web-Based Decision Tool to Improve Contraceptive Counseling for Women With Chronic Medical Conditions: Protocol For a Mixed Methods Implementation Study

**DOI:** 10.2196/resprot.9249

**Published:** 2018-04-18

**Authors:** Justine P Wu, Laura J Damschroder, Michael D Fetters, Brian J Zikmund-Fisher, Benjamin F Crabtree, Shawna V Hudson, Mack T Ruffin IV, Juliana Fucinari, Minji Kang, L Susan Taichman, John W Creswell

**Affiliations:** ^1^ Department of Family Medicine University of Michigan Ann Arbor, MI United States; ^2^ VA Center for Clinical Management Research Ann Arbor, MI United States; ^3^ Department of Health Behavior and Health Education University of Michigan Ann Arbor, MI United States; ^4^ Department of Internal Medicine University of Michigan Ann Arbor, MI United States; ^5^ Department of Family Medicine and Community Health Rutgers Robert Wood Johnson Medical School New Brunswick, NJ United States; ^6^ Department of Family and Community Medicine Penn State Milton S. Hershey Medical Center Hershey, PA United States

**Keywords:** contraception, mobile apps, birth control, primary care physicians, implementation science, decision support techniques, chronic disease, multiple chronic conditions, qualitative research

## Abstract

**Background:**

Women with chronic medical conditions, such as diabetes and hypertension, have a higher risk of pregnancy-related complications compared with women without medical conditions and should be offered contraception if desired. Although evidence based guidelines for contraceptive selection in the presence of medical conditions are available via the United States Medical Eligibility Criteria (US MEC), these guidelines are underutilized. Research also supports the use of decision tools to promote shared decision making between patients and providers during contraceptive counseling.

**Objective:**

The overall goal of the *MiHealth, MiChoice* project is to design and implement a theory-driven, Web-based tool that incorporates the US MEC (provider-level intervention) within the vehicle of a contraceptive decision tool for women with chronic medical conditions (patient-level intervention) in community-based primary care settings (practice-level intervention). This will be a 3-phase study that includes a predesign phase, a design phase, and a testing phase in a randomized controlled trial. This study protocol describes phase 1 and aim 1, which is to determine patient-, provider-, and practice-level factors that are relevant to the design and implementation of the contraceptive decision tool.

**Methods:**

This is a mixed methods implementation study. To customize the delivery of the US MEC in the decision tool, we selected high-priority constructs from the Consolidated Framework for Implementation Research and the Theoretical Domains Framework to drive data collection and analysis at the practice and provider level, respectively. A conceptual model that incorporates constructs from the transtheoretical model and the health beliefs model undergirds patient-level data collection and analysis and will inform customization of the decision tool for this population. We will recruit 6 community-based primary care practices and conduct quantitative surveys and semistructured qualitative interviews with women who have chronic medical conditions, their primary care providers (PCPs), and clinic staff, as well as field observations of practice activities. Quantitative survey data will be summarized with simple descriptive statistics and relationships between participant characteristics and contraceptive recommendations (for PCPs), and current contraceptive use (for patients) will be examined using Fisher exact test. We will conduct thematic analysis of qualitative data from interviews and field observations. The integration of data will occur by comparing, contrasting, and synthesizing qualitative and quantitative findings to inform the future development and implementation of the intervention.

**Results:**

We are currently enrolling practices and anticipate study completion in 15 months.

**Conclusions:**

This protocol describes the first phase of a multiphase mixed methods study to develop and implement a Web-based decision tool that is customized to meet the needs of women with chronic medical conditions in primary care settings. Study findings will promote contraceptive counseling via shared decision making and reflect evidence-based guidelines for contraceptive selection.

**Trial Registration:**

ClinicalTrials.gov NCT03153644; https://clinicaltrials.gov/ct2/show/NCT03153644 (Archived by WebCite at http://www.webcitation.org/6yUkA5lK8)

## Introduction

### Quality Gaps in Contraceptive Care

Access to family planning services to prevent unintended pregnancies is one of the leading health indicators for Healthy People 2020 [1]. Unintended pregnancies account for half of all US pregnancies [2] and are associated with adverse outcomes for women and children, such as maternal depression and low birth weight, respectively [3,4]. In 2008, US expenditures for live births resulting from unintended pregnancies were US $12.5 billion [5]. In 2014, the Centers for Disease Control and Prevention (CDC) and the US Office of Population Affairs jointly recommended an increase in the volume and quality of family planning services across all health care sectors, including primary care, to address this unmet public health need [6]. The Office of Population Affairs subsequently released contraceptive quality measures that assess the percentage of reproductive-aged fertile women who are provided moderately effective and highly effective contraceptive methods [7]. The National Quality Forum formally endorsed these measures in 2017, and the development of a patient-reported measure of contraceptive care is in progress [7]. There is a timely and critical need to disseminate and implement evidence-based interventions to meet these contraceptive quality measures and improve reproductive health outcomes.

### Implications for Women With Chronic Medical Conditions

Women with chronic medical conditions (eg, diabetes and hypertension) have a higher rate of pregnancy-related complications [8-11] and death compared with women without these conditions [12,13]. The most prevalent chronic medical conditions (hereafter called “chronic conditions”) among reproductive-age women have risen over the last 10 years and include obesity (24.7%), asthma (16.2%), high cholesterol (13%), hypertension (10%), and diabetes (2.9%) [14]. Expanded definitions of chronic conditions that include psychiatric conditions estimate that women with chronic conditions comprise up to 45% of reproductive age women seen in primary care [15,16]. Studies have raised concerns that adult women with chronic conditions are at greater risk for unplanned pregnancy [17] as they are more likely to not use any contraceptive method, underutilize the most effective methods, and rely upon the least effective methods compared with the general population of reproductive-aged women [18-21]. Women with chronic conditions are often prescribed medications that can cause fetal defects [22,23], and those who do not desire pregnancy should be offered contraceptive options. Contraceptive counseling should include an explanation of potential beneficial or adverse impact of a method on their conditions and interactions with ongoing drug therapy.

### Missed Opportunities and Barriers to Contraceptive Care in Primary Care

Women with chronic conditions most frequently see primary care providers (PCPs) for their health management [24]; these visits are windows of opportunity to address contraception within the context of ongoing medical care [25]. PCPs are well situated to address the contraceptive needs of women with chronic conditions, but the time constraints of office visits and incomplete provider knowledge are commonly cited barriers to doing so [26-28]. Although family planning is a required part of training for most PCPs, contraceptive knowledge is lower among PCPs compared with obstetrics and gynecology providers [27,28]; this is not surprising given the greater intensity of training and exposure to women’s health care among obstetrics and gynecology providers.

### Implementation of Evidence-Based Contraceptive Guidelines From the Centers for Disease Control and Prevention

In 2010, the CDC released the United States Medical Eligibility Criteria (US MEC), which was adapted directly from the World Health Organization’s MEC to meet the unique needs of US patients. The US MEC provides guidance to clinicians regarding the selection of contraceptive methods in the presence of specific chronic conditions (eg, seizures) and personal characteristics (eg, age) [29] and is revised on a continual basis. In 2011, the American Congress of Obstetricians and Gynecologists formally endorsed use of the US MEC for “clinicians providing family planning services for women, especially women with chronic conditions” [30] as an effort to promote national-level dissemination and implementation of evidence-based contraceptive practices. However, there is a significant gap between the US MEC and reported clinical recommendations, particularly with respect to the intrauterine device and the implant—the most effective long-acting reversible contraceptives (LARC) [26,28,31-34]. LARC methods are estrogen-free and safe for the vast majority of women, including those with conditions that may preclude the use of estrogen (eg, cardiac disease) [35-37]. It is critical to correct PCP misconceptions about LARC eligibility so that they do not unnecessarily prevent LARC use among women with chronic conditions, who are otherwise appropriate candidates [36], thus placing them at risk for unintended pregnancy.

### Evidence-Based Contraceptive Counseling With Electronic Decision Aids

Studies have shown that provider recommendations have a significant and positive impact on patient initiation and selection of a contraceptive method [38-40]. However, the provision of generic contraceptive information alone is insufficient. Prior literature highlights the importance of individualized contraceptive counseling [41-43] via a shared decision-making process [44-47], defined as an interactive process through which providers and patients communicate and arrive at a mutually agreeable decision [48]. Decisions aids are clinical tools designed to support patient-centered communication via shared decision making rather than provide paternalistic or generic information [41,49]. Prior contraceptive decision aids have been developed for use on electronic tablet or computer-based platforms across multiple geographic regions, practice settings, and patient populations and have been associated with improved patient involvement [50], decreased decisional conflict, increased patient knowledge [51], and increased contraceptive use [45,52]. Patients have reported numerous advantages to a Web-based platform over paper, including the interactive nature of the interface and the ability to compare contraceptive methods using filters and sorting options [53,54]. Furthermore, patients appreciated the use of a decision tool before a clinical visit to help them narrow down their contraception options and prepare questions for their providers [53].

### Rationale for Mixed Methods Study Design

The underlying rationale for collecting, integrating, and analyzing both qualitative and quantitative data are multifold: (1) quantitative data collected from self-administered survey items will provide descriptive statistics to allow for comparison with other practice settings and populations, (2) qualitative interviews will provide a deeper understanding of the lived experiences of women with chronic conditions and their primary care teams with respect to receiving and providing contraceptive services, respectively, (3) qualitative interviews provide an opportunity to immediately expand upon close-ended quantitative survey items that warrant further investigation [55], (4) leveraging the complementary nature of quantitative data and qualitative data maximizes our capacity to assess a broader range of theoretical constructs and contextual factors than if quantitative or quantitative methods were used alone [56], (5) collecting data via multiple methods (observations, interviews, surveys) improves the robustness and credibility of our findings [57].

### The Use of Theory and Implementation Science to Develop a Patient-Centered Intervention for Use in Usual Care Settings

To ensure the development of a patient-centered tool that explicitly upholds patient autonomy in decision making, we created a conceptual model that draws upon principles from reproductive justice theory and health behavior theories. This conceptual model will provide a preliminary prototype for the decision tool, which will be modified iteratively during this study phase. To develop an intervention that is contextualized for use in real-world clinical practices, our study design is informed by implementation science, an emerging field of methods and approaches that address the challenges of implementing health interventions in usual practice settings.[58] We use selected constructs from 2 frameworks commonly used in implementation science: the Consolidated Framework for Implementation Research (CFIR) [59] and the Theoretical Domains Framework, to guide data collection and analysis on the practice and provider level, respectively.

The overall goal of this mixed methods implementation project, the *MiHealth MiChoice study*, is to design and implement a theory-driven, Web-based contraceptive decision tool that can be accessed on an electronic tablet or computer before a clinical visit by women with chronic conditions who are seen in primary care settings. The feature of this tool that sets it apart from prior decision aids is that it will be tailored to factor in the personal preferences and medical history of a specific individual in a manner that also reflects evidence-based guidelines. The development and testing of this tool will occur over 3 phases (a predevelopment, development, and testing phase). The aim of this study protocol for phase 1 is to identify multilevel contextual factors that should drive the design and implementation of the contraceptive decision tool and explain subsequent study outcomes [60].

## Methods

### Overall Study Design

**Figure 1:**
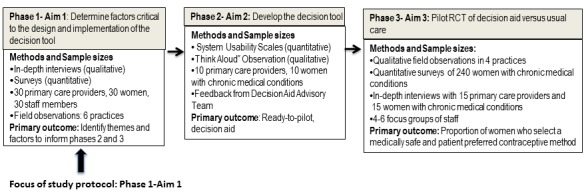
Multiphase mixed methods design. PCP: primary care provider; RCT: randomized controlled trial; CC: chronic condition.

This mixed methods implementation study consists of 3 phases. This protocol focuses on phase 1, which is to identify the most critical patient-, provider-and practice-level factors that should inform the design and implementation of the decision-support tool. In phase 2, we will work with an expert health informatics team and an advisory council comprising patients, providers, and decision aid experts to build the Web-based decision tool that is accessible via a secured weblink from a computer or handheld tablet. Findings from phase 1 and a novel conceptual model (described in Figure 1 below) will inform iterative prototypes of the decision tool. In phase 3, the decision tool will be compared with usual care in a randomized controlled trial. We have received ethics approval for this study protocol by the University of Michigan Institutional Review Board (HUM00128060).

### Phase 1 Study Design

This is a convergent mixed methods design phase that focuses primarily on qualitative data collection and analysis with concurrent quantitative data collection and analysis. We will conduct semistructured, qualitative interviews of patients, PCPs, and practice staff members. All study participants will complete quantitative written surveys or electronic surveys via Qualtrics (Qualtrics, Provo, UT) before their qualitative interviews. The quantitative survey items complement the qualitative interview guide with the goal of obtaining maximal depth and breadth of understanding of each construct. Semistructured field observations in practices and collection of practice artifacts (eg, clinical protocols and patient intake forms) will further diversify our data sources and optimize data triangulation, which in turn will reduce the risk of systematic biases based on reported behaviors and experiences alone [61].

### Sampling Strategy, Eligibility, and Recruitment

#### Practices

To balance similarity and variation across practices, providers, and patients, we will use a combination of purposeful sampling techniques as described by Palinkas and colleagues [62]. Eligible practices include practices that identify as family medicine, internal medicine, internal medicine-pediatric, or any combination of these. First, we will use criterion sampling to select individuals who have experiences that are relevant to the phenomenon of interest [62]; for this study, we aim to select practices for which a contraceptive intervention is both clinically relevant and feasible to implement. Because LARC methods (intrauterine device and implant) are the most effective reversible methods and medically appropriate for the vast majority of women with chronic conditions [36,37,63], we will recruit practices that either provide LARC or assist with referrals for LARC. Therefore, eligible practices must have: (1) one or more providers who currently offer prescription contraception (eg, oral contraceptive pills), and (2) informal or formal processes to refer patients who desire LARC methods to another site *or* provide LARC methods on site. To achieve maximum variation in practice attributes that are associated with variations in contraceptive practice [31,64], clinical sites will be sampled to reflect a range of location (urban, suburban, and rural practices), a balance between private practices and practices with federal designations (federally qualified health centers, rural health center, medically underserved areas), and diversity in the racial/ethnic background of patients. Thus, maximum variation sampling aims to achieve breadth in sampling and can highlight differences between practices. To complement this approach, we will also use snowball sampling such that participating practices suggest other practices for recruitment; this approach tends to select practices that share characteristics and thus will help to achieve depth of understanding of similar practices [62]. We will recruit practices through The Great Lakes Research Into Practice Network, a statewide practice-based research network that is recognized by the Agency for Healthcare Research and Quality [65].

#### Sample Size

To achieve the depth and variation in practices as described above, we aim to enroll 6 clinical sites. For individual qualitative interviews, prior literature has documented that 6 to 12 interviews per homogeneous group provide sufficient qualitative data to reach saturation, the point at which analysis produces no new information, or disconfirming or confirming evidence [55,66]. On the basis of the sampling strategy described, we aim to enroll 30 patients, 30 PCPs, and 30 staff members (nurses, medical assistants, and administrative staff). Our definitions of homogenous groups are summarized below and in Multimedia Appendix 1. Purposeful sampling will be driven by this matrix such that the perspectives of individuals in each category are represented in qualitative interview data with the goal of data saturation. We expect this category to evolve based upon patient population characteristics in recruited practices.

#### Practice Members (Primary Care Practices and Staff)

Eligible practice members must be aged 18 years or older, English-speaking, able to give informed consent, and be indirectly or directly involved with patient care. PCPs must be physicians, nurse practitioners, physician assistants, or certified nurse midwives, who currently provide preventive health and management of chronic conditions to reproductive-aged women [62]. To complement criterion sampling as described above, we seek maximal variation [62] in PCPs’ contraceptive practices and will sample individuals who: (1) do not provide prescription contraception (eg, oral contraceptive pills), (2) provide prescription contraception but *do not* insert LARC devices, or (3) provide prescription contraception *and* insert LARC devices. For practice staff members other than PCPs, we aim to gather staff perspectives regarding contraceptive services and interventions that may differ based upon their primary responsibilities and context of patient interaction: (1) director or manager (clinical director, administrative director, nurse manager), (2) work with PCPs during clinical visits (nurses, medical assistants, licensed practical nurses), and (3) other services (social work, complex care management, pharmacist, behavioral counselor). A designated practice liaison (eg, medical director, office manager) will assist the study team to identify eligible practice members and extend invitations for study participation.

#### Patients

Eligible patients must be women aged 18 to 50 years, fertile, English-speaking, and able to provide informed consent. They must also meet at least one of the following criteria: (1) a documented medical condition or multiple medical conditions being *actively managed* (on medication or requiring at least 2 visits a year), (2) a documented past medical condition or multiple medical conditions which would pose a significant risk to health during pregnancy (eg, past lung clot), or (3) current use of any drugs that are Pregnancy Category D or Category X medications. This definition expands upon guidance provided by the Department of Health and Human Services [67] as well as informed by pilot interviews with 15 PCPs (unpublished data). To obtain a range of perspectives, we aim to sample approximately equal numbers of women in the following groups that consist of conditions commonly encountered in primary care [68], conditions that frequently coexist together, or are managed with similar behavioral approaches and medications. Furthermore, we will focus on conditions for which there is evidence-based guidance regarding contraceptive selection in the CDC US MEC [69]. The following groups are described in detail in Multimedia Appendix 1: (1) psychiatric conditions, (2) metabolic and endocrine conditions, and (3) neurologic conditions. We will include an *other* group to capture women with less common conditions that nevertheless have a significant impact on pregnancy-related morbidity and mortality and contraceptive eligibility. The principal investigator (JW), who is a primary care specialist and a family planning expert, will review each participant’s medical history and medications, in conjunction with the designated practice liaison, to ensure that eligibility criteria have been met.

The designated practice liaison in each practice will assist the study team in identifying patients who meet the eligibility criteria. With the permission of their PCPs, recruitment letters will be sent to potentially eligible patients followed up by up to 3 phone calls.

### Context Assessment Frameworks to Guide Implementation (Practice and Provider Level)

Dehlendorf and colleagues recently described the development and testing of a tablet-based contraceptive decision aid that underwent rigorous cognitive testing [70] among patients in a safety net clinic. Our adaptation of this decision aid model will be sensitized by the application of our data to selected constructs from the CFIR, a typology of 5 major domains and associated constructs to assess context [59]. We chose CFIR because it identifies constructs at the practice and provider level that are universally relevant to successful implementation of a new intervention in clinical practice and can be tailored to the context of contraception. Furthermore, we anticipate that the use of CFIR will facilitate the collection of qualitative and quantitative data in a harmonized and efficient manner. Because the proposed intervention integrates clinical decision support for individual health providers, we also adapted constructs from the Theoretical Domains Framework to systematically identify determinants of clinical behavior change among PCPs [71]. We created a mixed methods theory-data matrix (see Multimedia Appendices 2-4), CSIR constructs and definitions by Damschroder et al [59]) to summarize how qualitative data (derived from practice observation, artifacts, and interviews) and quantitative data (derived from surveys) map to CFIR and Theoretical Domains Framework constructs (see Multimedia Appendices 2-4).

**Conceptual Model to Guide Development of the Decision Tool (Patient-Level)**

**Figure 2:**
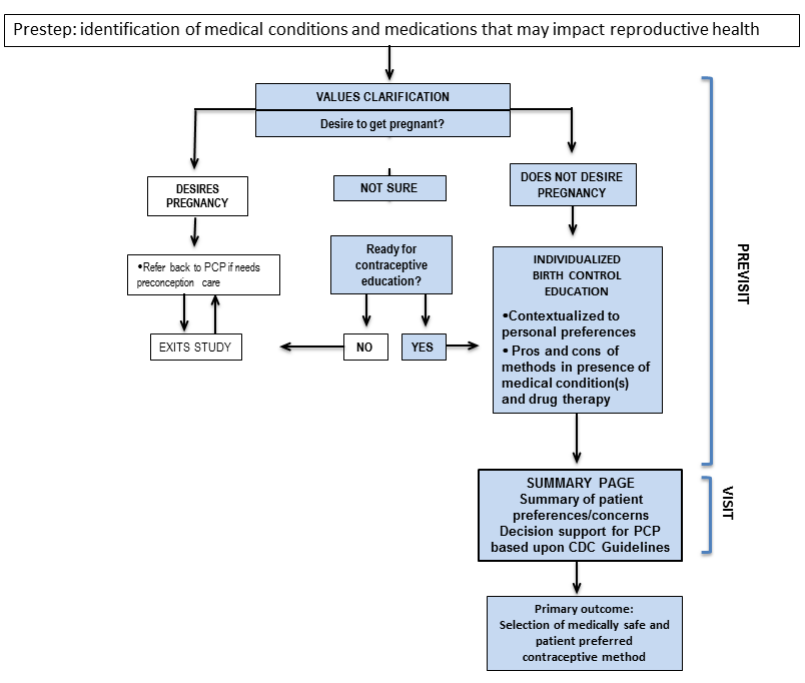
Conceptual model to guide design of decision tool. PCP: primary care provider; CDC: Centers for Disease Control and Prevention.

In a systematic review, Wyatt and colleagues identified 32 unique characteristics among 19 decision aids and classified them into 4 overarching categories: method effect, mechanistic, social/normative, and practical [72]. Among these attributes, studies have shown that women prioritize knowledge regarding mechanism of action [44], contraceptive effectiveness, safety, and side effects [73]. Using the tablet-based decision aid described by Dehlendorf as the prototype model [70], we will customize the above high-priority attributes for this patient population. Using a drop-down menu function, women will first provide their basic health history, including age, smoking status, medical conditions, and medications. We will then elicit patient preferences, guided by a conceptual model that incorporates a synthesis of constructs from reproductive justice theory, behavioral health theories, and evidence-based counseling techniques such as motivational interviewing and values clarification (Figure 2). One of the central tenets of reproductive justice is that people should be equally afforded the right to have a child and parent as well as the right to not have a child [74]. In accordance with this principle, we assert that a patient-centered contraceptive tool must be designed to prevent unconscious or conscious reproductive coercion, particularly toward individuals from marginalized communities. This concern is based upon the disturbing legacy of compulsory sterilization programs that targeted women of color, poor women, women with disabilities, and immigrant women in multiple US states throughout the twentieth century [75] and even as recently as 2010 in California [76]. Therefore, the model explicitly avoids presumptions about the patient’s feelings regarding pregnancy and childbearing and starts with a values clarification [77] question by asking the patient about her current feelings regarding pregnancy and parenting.

Adapting a principle from the transtheoretical model [78], the patient will receive recommendations that are “matched” to her current pregnancy desires. If she does not want to get pregnant *and* indicates she is ready for contraceptive education, she will proceed through a responsive algorithm based upon her personal preferences, concerns, and prior contraceptive experiences. Constructs from the health beliefs model [79] will be operationalized to provide her information regarding the potential impact of her chronic condition on her reproductive health and vice versa, as well as individualized pros and cons of different contraceptive methods. The decision tool will summarize her preferences for methods, concerns she may want to discuss with her PCP, and embed clinical decision support for the PCP based upon the US MEC Guidelines (eg, patient has severe diabetes and should not use estrogen). This information will be made available in paper or electronic form to serve as a template for provider-patient discussion during the office visit.

If the patient indicates she desires pregnancy, she is advised to return to her PCP for preconception counseling and exits the study at this time. If the patient expresses ambivalence regarding pregnancy, the decision aid assesses if she is ready for contraceptive education, and if so, she proceeds through the contraceptive algorithm as outlined above.

### Data Collection

Trained research assistants (RA) will spend 2 to 3 days at each clinical site to collect practice-specific data using a Practice Environment Template (PET), a semistructured checklist adapted from prior work by Crabtree and colleagues [80] and Jaén and colleagues [81]. The PET cues the RA to record observations of routine office activities, with a focus on clinical flow and processes relevant to contraceptive services. These data will provide a more complete and nuanced description of “what is happening on ground” and identify data that participants may not report on surveys or during interviews. The designated practice liaison will complete the Practice Information Form, a 29-item survey, also modified from prior work [80,81], that consists of multiple choice and open response items regarding practice demographics, pay structure, preventive and reproductive health services offered, and contraceptive methods offered.

All participants (staff, PCPs, and patients) will fill out a written or electronic (via Qualtrics, Provo, UT) quantitative survey before the face-to-face in-depth interview. There are 3 surveys: (1) a 34-item Patient Survey, (2) a 19-item Provider Survey (for PCPs), and (3) a 13-item Staff Survey (for practice members other than PCPs). Participants will be interviewed in a quiet, private space designated by each practice. Interviews are audiotaped with the participants’ permission and informed consent. We will conduct “member checking” with participants who agree to be contacted after the interview. This qualitative technique, also referred to as respondent validation, helps improve the accuracy, credibility, and transferability of research findings as well as empower participants to verify or modify the final interpretations of the data [61]. Member-checking should be undertaken with caution to minimize the risk of participant discomfort and ensure anonymity [82]. Therefore, we will share general themes and aggregated group data rather than specific quotes from individuals. All participants who complete an in-depth interview will receive a US $30 gift card as a token of appreciation for their time.

### Data Analysis

All interview audiotapes will be transcribed verbatim. Qualitative analysis is an iterative process during which investigators go through cycles of reading, summarizing, and re-reading data [83,84]. The qualitative team is composed of 4 individuals from different professional backgrounds and research disciplines, including family medicine, dentistry, epidemiology, and health behavior. Though all team members identify as female, they vary in age, sexual orientation, religious background, and race/ethnicity. The interview transcripts will be uploaded and organized using MAXQDA software (VERBI GmbH, Berlin, Germany Version 12.3.1). We will conduct analysis through a series of iterative steps adapted from techniques described by Marshall and Rossman [85]. First, each team member will review several transcripts independently and code the content of each transcript. Because our research design is driven by predetermined theoretical constructs and research aims, our initial coding will be done with a theory-generated code template [86]. In vivo coding will also occur as new themes emerge from the interviews [85]. The team members will discuss, compare, and reconcile differences in coding and create a consensus code template, which will then be used to code the remainder of transcripts. Analysis of semistructured observations and practice artifacts proceeds in similar manner as described for interview transcripts. Themes and patterns will be identified and synthesized, using the preidentified theoretical constructs as a guide (Multimedia Appendices 2-4), as well as new codes and themes as they emerge. To increase the trustworthiness of our qualitative findings, we will triangulate our qualitative findings on multiple levels [54]: (1) methodological triangulation, by comparing and integrating with quantitative survey data, (2) data triangulation, by comparing and contrasting data obtained via interviews, surveys, observations, and artifacts, and (3) theoretical triangulation, by gathering multiple perspectives of the same phenomenon (patient-, provider-, practice-level perspectives). Data collection continues until saturation is reached, or until we no longer identify new or disconfirming or confirming data [84] with respect to the original research aim. Quantitative survey data will be summarized with simple descriptive statistics (frequencies, means, and SDs). We will conduct bivariate analyses with Fisher exact test to explore the following relationships: (1) demographic traits of providers and their contraceptive recommendations and practices, (2) practice attributes and providers’ contraceptive recommendations and practices, (3) the presence of different chronic conditions and current pregnancy desires among women, and (4) the presence of different chronic conditions and current contraceptive use among women. As described by Fetters [60], we will merge the quantitative and qualitative strands of data by identifying content from both datasets to compare, contrast, and synthesize. A final interpretation will summarize to what extent and how the results from the qualitative and quantitative data contribute to the identification of patient-, provider-, and practice-level factors that will then shape the design and implementation of the decision tool.

## Results

Enrollment of patients and providers in community-based primary care practices in Michigan is underway. Upon completion of this first study phase, the findings will be used to inform design of the contraceptive decision tool for testing in a future randomized controlled trial. The results of this study will be published in a peer-reviewed journal and presented at scientific conferences.

## Discussion

The study design and proposed intervention have several strengths. First, we are collecting multilevel qualitative and quantitative data to gain a comprehensive and deep understanding of the experiences of and interactions among patients, providers, and staff. Constructs from implementation science theory and behavioral health theories drive data collection and analysis. To organize this large volume of data, we employ a rigorous mixed methods design and data integration procedures. A potential weakness of this study is that practices are limited to Michigan, which has a lower prevalence of ethnic and racial minorities than more populous states. However, we will recruit practices that have greater representation of underrepresented groups to mitigate this concern. We also anticipate challenges associated with practice-based research, including efficient recruitment and coordination of research practices with multiple practices outside our institution. The support of a locally based and established state-wide primary care network and previously established relationships between our institution and community partners will be critical to these processes.

This protocol describes the first phase of a multiphase design and implementation of a theory-driven intervention that incorporates customized decision tool attributes and embeds targeted US MEC recommendations to meet the contraceptive needs of women with chronic medical conditions in primary care settings. The study findings will provide critical knowledge regarding the feasibility and best approaches to implement the intervention in real-world primary care settings with the goal of promoting shared decision-making and evidence-based guidelines.

## Appendix

Multimedia Appendix 1 Qualitative sampling matrix.

Multimedia Appendix 2 Mixed methods theory data matrix: mapping data to Consolidated Framework for Implementation Research (CFIR) inner setting constructs.

Multimedia Appendix 3 Mixed methods theory data matrix: mapping data to Consolidated Framework for Implementation Research (CFIR) outer setting and intervention constructs.

Multimedia Appendix 4 Mixed methods theory data matrix: mapping data to Theoretical Domains Framework (TDR) constructs.

## Abbreviations

CDC: Centers for Disease Control and Prevention
CFIR: Consolidated Framework for Implementation Research
LARC: long-acting reversible contraceptives
PCP: primary care provider
PET: Practice Environment Template
US MEC: United States Medical Eligibility Criteria
