# Supplementary material for: A Web-Based Decision Tool to Improve Contraceptive Counseling for Women With Chronic Medical Conditions: Protocol For a Mixed Methods Implementation Study
Source: JMIR Res Protoc. 2018 Apr 18;7(4):e107. doi: 10.2196/resprot.9249 (PMC5932336; doi:10.2196/resprot.9249)
Supplement: Multimedia Appendix 1 [file resprot_v7i4e107_app1.pdf]

Table 1. Qualitative sampling matrix: patients, primary care providers, and practice staff members.

Patients	Primary care providers	Practice staff members
**Psychiatric conditions:** depression, anxiety, attention deficit hyperactivity syndrome, bipolar disease, use of controlled substances, substance abuse.	**No contraceptive Rx[b]**: providers who do not provide any contraception that requires a prescription.	**Director and managers:** staff who have supervisory responsibilities and oversight of clinical and financial aspects of the practice (eg, clinical director, medical director, nurse manager).
**Metabolic and endocrine conditions:** diabetes, high blood pressure, high cholesterol, morbid obesity, thyroid problems.	**Contraceptive Rx[b], No LARC[c]:** providers who provide contraceptive prescriptions (eg, pill, patch, ring, shot) but do not insert and remove long-acting reversible methods (LARC)[c].	**Work with PCPs[a]**: staff who assist PCPs during clinical visits and directly interact with patients (eg, nurses, medical assistants, licensed practical nurses).
**Neurologic conditions:** epilepsy, migraines, chronic pain, multiple sclerosis.	**Contraceptive Rx and LARC[c]**: providers who provide contraceptive prescriptions (eg, pill, patch, ring, shot) AND insert and remove long-acting reversible methods (LARC)[c].	**Other services:** other staff who may interact with women with chronic conditions (eg, social workers, pharmacists, behavioral counselors, complex care managers).
4.Other: clotting disorders, bleeding disorders, cancer, HIV/AIDs, rheumatologic disorders, gastrointestinal disorders.		

Notes:
[a]PCPs: primary care providers.
[b]Rx: prescription.
