# Supplementary material for: A Web-Based Decision Tool to Improve Contraceptive Counseling for Women With Chronic Medical Conditions: Protocol For a Mixed Methods Implementation Study
Source: JMIR Res Protoc. 2018 Apr 18;7(4):e107. doi: 10.2196/resprot.9249 (PMC5932336; doi:10.2196/resprot.9249)
Supplement: Multimedia Appendix 2 [file resprot_v7i4e107_app2.pdf]

Table 2a. Mixed Methods Theory Data Matrix: Mapping data to Consolidated Framework for Implementation Research (CFIR) Inner Setting Constructs

Adapted Constructs	Quantitative Data	Data Source & Items	Qualitative Data	Data Source & Items
**INNER SETTING**				
<u>**Structural characteristics**</u>: age, maturity of the practice, external and internal infrastructure, clinical processes that affect women' experience of care, with focus on women's health/reproductive health	Rural, suburban, urban location?<br>Any special federal designations?<br>Clinical specialties represented<br>How many providers?<br>Who owns the practice?<br>How old is the practice?<br>How many practice sites?<br>Patient demographics<br>Use of EMR, web portals?<br>Patient scheduling, access, volume	PIF 1<br>PIF 2<br>PIF 3<br>PIF 4<br>PIF 5<br>PIF 9<br>PIF 10<br>PIF 11-14<br>PIF 13<br>PIF 15-20	Location<br><br>Waiting room observations<br><br>Clinical flow<br><br>Physical structure of building<br>Exam rooms and equipment<br><br>Interior structure	PET
<u>**Networks & Communications**</u>: networks and communications that facilitate practice research engagement, population management of women with medical conditions, access to birth control and LARC	Is the practice part of a Research Network?	PIF 8	Does your practice have system wide prompts or workflows to promote preventive care? Please give an example.	PG 1
	System to track and manage patients with specific conditions?	PIF 24	Are there system prompts for medications that may cause fetal defects? What are your thoughts about that?	PG 1<br>SG 1
	How would you manage/refer a patient who wants LARC?	PS 14	You noted that you would recommend X if a patient requested an IUD or implant. Can you walk me through how this typically proceeds?	PG 8

**<u>Learning climate</u>**: degree to which practice climate is conducive to change process based upon team dynamics and relationships			Nature of relationships and interactions among practice team, reactions to stress, team planning	PET
Implementation Climate **<u>Relative priority:</u>** current clinical services and stakeholders' attitudes that reflect relative priority of family planning in the practice	Which services are offered in this practice? (cervical cancer screening, miscarriage care, prenatal services, reproductive health services)	PIF 21, SS 6	What do the providers consider to be the top priority health topics to address during a well woman exam?	SG 4
			There are often competing demands during a well exam. If there is limited time, which health topics are most important for you to address with Mrs. Gold (clinical vignette 1)	PG 2
	Which contraceptive options are offered in this practice?	PIF 22, SS 8	Studies show that birth control is not routinely addressed during the well woman visit. Based upon our experience what may contribute to this finding?	SG 4
	Does your practice have clinical protocols or policies for prescribing birth control? For providing LARC?	PIF 23, SS 9	How do patients get birth control prescriptions or refills? Can you give me an example?	SG 7
	Does your practice offer LARC insertion and/or referral for LARC insertion?	PIF 25-29, SS 10-11	What happens if a patient requests an IUD? An implant?	SG 8

<u>Notes for Tables 2a and 2b.</u> Practice Information Form (**PIF**): 29 item survey with multiple choice and open response items completed by practice liaison regarding key practice characteristics; Practice Environment Template **(PET)**: Semi-structured template for practice observations; Provider Survey (**PS**) 19 item survey with multiple choice questions; Provider Interview Guide (**PG**): Semi-structured 13 question interview guide with a clinical vignette; Staff Survey (**SS**) 13 multiple choice items; Staff Interview Guide (**SG**); Semi-structured structured 9 question interview guide; **LARC** (long-acting reversible contraceptives); Consolidated Framework for Implementation Research (**CFIR**) constructs and definitions: http://www.cfirguide.org/constructs.html
