# Supplementary material for: A Web-Based Decision Tool to Improve Contraceptive Counseling for Women With Chronic Medical Conditions: Protocol For a Mixed Methods Implementation Study
Source: JMIR Res Protoc. 2018 Apr 18;7(4):e107. doi: 10.2196/resprot.9249 (PMC5932336; doi:10.2196/resprot.9249)
Supplement: Multimedia Appendix 3 [file resprot_v7i4e107_app3.pdf]

Table 2b. Mixed Methods Theory Data Matrix: Mapping data to Consolidated Framework for Implementation Research (CFIR) Outer Setting and Intervention Constructs

**Adapted Constructs**	**Quantitative Data**	**Data Source & Items**	**Qualitative Data**	**Data Source & Items**
**OUTER SETTING**				
**Patient needs and resources**: the extent to which women's family planning needs are known and prioritized	How prepared do you feel this practice is to address the birth control needs of women? (very prepared, somewhat prepared, somewhat unprepared, very unprepared)	SS 13	You noted this practice is X prepared to address birth control what led you to this answer? What challenges does this practice face? What particular assets/resources does this practice have?	SG 9
**Cosmopolitanism:** the degree to which the practice is networked with other organizations	Is the practice is part of a Research Network?	PIF 8		
**External policy and incentives:** Engagement in models of practice reform and payment reform, competing demands of external programs	Is this practice part of an Accountable Care Organization (ACO)?	PIF 6		
	Is this practice recognized as a Patient Centered Medical Home (PCMH)?	PIF 7		
**INTERVENTION CHARACTERISTICS**				
**Evidence strength and quality**: Providers' current use and perceptions evidence-based contraceptive guidelines (US MEC) and other contraceptive resources	Have you ever applied the US MEC guidelines in your practice?	PS 18	Studies have shown that the US MEC is not widely used. What do you think could be explanations for this? What features are missing? What features do you like? (US MEC Chart and phone app shown to provider)	PG 11

	What professional resources have you used to inform your contraceptive counseling and care? (colleagues, online resources, phone apps, clinical guidelines, other)	PS 15	Can you give me an example of what you have used this resource? Which one of these do you prefer? Why?	PG 10
**Relative advantage:** Providers' perceptions of the features that would be most advantageous for a contraceptive intervention			What resource format would be most helpful to improve point of care for contraceptive counseling (paper, EMR, tablet, phone)?	PG 12
			One way patients can get information is through self-directed decision aids. What do you think? What features of a decision aid do you think are critical to be useful for patients?	PG 13
**PROCESS**				
**Champions:** Identification of current or possible women's health champions in practices	Are there any "women's health champions" in this practice?	SS 12	Can you give me an example of how this person is a women's health champion?	SG 9

Notes: Practice Information Form (**PIF**): 29 item survey with multiple choice and open response items completed by practice liaison regarding key practice characteristics; Practice Environment Template **(PET)**: Semi-structured template for practice observations; Provider Survey (**PS**) 19 item survey with multiple choice questions; Provider Interview Guide (**PG**): Semi-structured 13 question interview guide with a clinical vignette; Staff Survey (**SS**) 13 multiple choice items; Staff Interview Guide (**SG**); Semi-structured structured 9 question interview guide; **LARC** (long-acting reversible contraceptives); Consolidated Framework for Implementation Research (**CFIR**) constructs and definitions: http://www.cfir-guide.org/constructs.html
