# Supplementary material for: A Web-Based Decision Tool to Improve Contraceptive Counseling for Women With Chronic Medical Conditions: Protocol For a Mixed Methods Implementation Study
Source: JMIR Res Protoc. 2018 Apr 18;7(4):e107. doi: 10.2196/resprot.9249 (PMC5932336; doi:10.2196/resprot.9249)
Supplement: Multimedia Appendix 4 [file resprot_v7i4e107_app4.pdf]

Adapted Constructs	Quantitative Data	Data Source & Items	Qualitative Data	Data Source & Items
**<u>Knowledge</u>:** familiarity with CDC evidence based guidelines for contraceptive use in women with selected characteristics and medical conditions	Assuming a patient requests an IUD, would you recommend this method in the following scenarios: (less than 20 years old, has never been pregnant, taking medications for well controlled hypertension, taking insulin for diabetes with retinopathy)	PS 16	Clinical Vignette 1:<br>Women with diabetes presents for well woman visit: “Tell me how you would typically approach this visit…”<br><br>Clinical Vignette 1: Same patient presents for chronic care visit: “Tell me how you would typically approach this visit.”<br>[follow up prompts regarding approach to family planning]	PG 1, 5
	Assuming a patient requests an oral contraceptive pill with estrogen, would you recommend this method in the following scenarios: (never had a pap smear, is a 25 year old smoker, taking medications for well controlled hypertension, taking insulin for diabetes with retinopathy)	PS 17		
	Are you aware of the US MEC, a clinical resource to guide the selection of contraceptive methods based upon patient characteristics and medical conditions?	PS 18		
**<u>Skills:</u>** current scope of contraceptive practice, current approach to contraceptive care for women with medical conditions	Please indicate if you prescribe the following contraceptive methods/ insert the following contraceptive devices/refer women who request contraceptive devices	PS 11-14		
**<u>Beliefs about capabilities</u>**: self-perception of ability to deliver contraceptive care	Please indicate if you feel prepared to discuss the following contraceptive methods with a patient: (list of all reversible methods)	PS 11	What information would you need to feel more prepared to discuss these methods?	PG 6
			Are there clinical situations/medical conditions that are more challenging than others?	PG 7

<u>Notes</u>: Provider Survey (**PS**) 19 item survey with multiple choice questions; Provider Interview Guide (**PG**): Semi-structured 13 question interview guide with a clinical vignette; IUD= Intrauterine device
